# Sex differences in MASLD-related atherosclerosis: plaque phenotype, vascular dysfunction, and cardiovascular outcomes

**DOI:** 10.1186/s13293-026-00952-3

**Published:** 2026-07-07

**Authors:** Mohamad Jamalinia, Ralf Weiskirchen, Amedeo Lonardo

**Affiliations:** 1 Gastroenterohepatology Research Center, Shiraz University of Medical Sciences, Shiraz, Iran; 2 Institute of Molecular Pathobiochemistry, Experimental Gene Therapy and Clinical Chemistry (IFMPEGKC), RWTH University Hospital Aachen, Aachen, Germany; 3 Independent Researcher, 41100 Modena, Italy

**Keywords:** Atherosclerotic cardiovascular disease, Coronary artery calcium, Cardiovascular imaging, Coronary artery disease, Endothelial dysfunction, Gender differences, MASLD, Myocardial infarction, Plaque phenotype, Sex differences

## Abstract

**Background:**

Metabolic dysfunction-associated steatotic liver disease (MASLD) is a major risk factor for atherosclerotic cardiovascular disease (ASCVD). Emerging evidence suggests that the vascular manifestations of MASLD differ substantially between men and women. We therefore hypothesized that the mechanisms, plaque phenotypes, and clinical implications of MASLD-related ASCVD are sex-specific.

**Methods and results:**

In this narrative review, we searched PubMed/MEDLINE for studies published up to April 2026 and synthesized current evidence regarding sex differences in MASLD-related atherosclerosis. Available data support a sex-specific vascular paradigm in MASLD. In men, MASLD is more strongly associated with calcified and obstructive coronary atherosclerosis. In contrast, women with MASLD more frequently exhibit non-calcified atherosclerotic phenotypes and functional vascular abnormalities (e.g., endothelial dysfunction, arterial stiffness, and coronary microvascular dysfunction). Importantly, despite lower coronary artery calcium (CAC) burden, women with MASLD experience a disproportionately greater risk of adverse cardiovascular events. Therefore, calcification-centered assessment may inadequately capture ASCVD risk in women with MASLD by failing to identify non-calcified plaque and functional vascular abnormalities. These sex differences are likely mediated by interacting pathobiological and socio-cultural factors, including sex hormones and menopausal transition, adipose tissue distribution, immune-metabolic and inflammatory profiles, genetic susceptibility, and gender influences.

**Conclusion:**

MASLD is associated with distinct sex-specific cardiovascular phenotypes that have important clinical implications for ASCVD risk assessment and prevention. Women with MASLD may develop clinically significant ASCVD despite lower CAC burden, supporting a sex- and menopause-aware approach to cardiovascular evaluation. In selected high-risk women, complementary use of multimodality imaging beyond CAC may improve risk stratification and facilitate earlier detection of clinically relevant cardiovascular disease.

**Supplementary Information:**

The online version contains supplementary material available at https://doi.org/10.1186/s13293-026-00952-3.

## Highlights

The vascular manifestations of metabolic dysfunction-associated steatotic liver disease (MASLD) represent a leading cause of mortality and substantially differ between men and women.

Men with MASLD more commonly exhibit calcified and obstructive coronary atherosclerosis; however, women with MASLD more frequently demonstrate non-calcified plaque, endothelial dysfunction, arterial stiffness, diffuse vascular disease, and coronary microvascular dysfunction.

Despite lower coronary artery calcium burden, women with MASLD may experience disproportionately greater risk of adverse cardiovascular events.

Clinicians and investigators should adopt a sex- and menopause-aware approach for atherosclerotic cardiovascular disease (ASCVD) risk evaluation in MASLD.

In selected women at high risk of coronary artery disease, complementary use of multimodality imaging beyond CAC may improve risk stratification and facilitate earlier detection of clinically relevant ASCVD.

**Supplementary Information:**

The online version contains supplementary material available at https://doi.org/10.1186/s13293-026-00952-3.

## Introduction

Metabolic dysfunction-associated steatotic liver disease (MASLD), formerly known as nonalcoholic fatty liver disease (NAFLD), has become one of the most prevalent chronic liver diseases worldwide and represents a growing global health challenge [1, 2]. Beyond its hepatic consequences, MASLD is increasingly recognized as a multisystem disorder with substantial extrahepatic complications [3]. Among these, cardiovascular disease (CVD) is the leading cause of morbidity and mortality in individuals with MASLD [4]. Strong evidence indicates close associations between MASLD and subclinical atherosclerosis, coronary artery calcification (CAC), adverse coronary plaque characteristics, myocardial infarction, stroke, and cardiovascular mortality [5–7]. Importantly, these associations often persist independently of traditional CVD risk factors, suggesting that MASLD itself contributes to the development and progression of atherosclerotic cardiovascular disease (ASCVD) [5–8].

Accumulating data indicate that the relationship between MASLD and ASCVD differs substantially by sex [9, 10]. For example, men generally develop MASLD and coronary artery disease (CAD) earlier in life and more commonly exhibit calcified and obstructive atherosclerotic plaque [10–12]. In contrast, women tend to develop MASLD later in life and more frequently demonstrate non-calcified or mixed plaque phenotypes and non-obstructive CAD [10–12]. However, female-specific life stages, including puberty, pregnancy, premenopausal, and menopausal transitions, are each associated with distinct risks for the development of ASCVD [13]. Furthermore, the relative CVD risk associated with MASLD may be greater in women than in men [6, 14]. Socio-cultural differences between men and women (i.e., gender differences) may also influence MASLD-related ASCVD and contribute to differences in CAD risk between the sexes [11, 15].

Previous reviews have largely addressed sex differences in either atherosclerosis or MASLD, but not their intersection. Atherosclerosis-focused literature describes sex-specific differences in plaque burden, composition, calcification, and vulnerability, whereas MASLD-focused reviews primarily emphasize hepatic pathogenesis, hormonal regulation, and cardiometabolic risk [13, 16, 17]. As a result, how biological sex modifies MASLD-related atherosclerotic remodeling remains insufficiently defined. Given that plaque phenotyping is central to ASCVD risk stratification and prevention by linking upstream metabolic dysfunction to downstream cardiovascular events, this knowledge gap is clinically relevant [18]. We assumed the biologically plausible hypothesis that the mechanisms, plaque phenotypes, and clinical implications of MASLD-related ASCVD are sex-specific. Therefore, in this narrative review, we synthesize current evidence on sex differences in MASLD-related atherosclerosis, with a focus on plaque phenotype, vascular imaging, and subsequent cardiovascular outcomes. We also provide a critical appraisal of the principal limitations of the existing literature and delineate future research priorities to address these knowledge gaps.

## Method

A literature search was performed in PubMed/MEDLINE for studies published up to April 2026 (Supplementary Table 1) to identify original studies that evaluated the sex-specific association between MASLD and ASCVD, coronary plaque burden or phenotype, vascular calcification, endothelial dysfunction, or cardiovascular outcomes. Due to the narrative nature of this review, formal quantitative meta-analysis or structured risk-of-bias assessment was not performed. Instead, the extracted evidence was qualitatively synthesized, with a focus on sex-specific differences in plaque characteristics and cardiovascular outcomes in individuals with MASLD.

## Pathophysiology of MASLD-related atherosclerosis with sex-specific modulation

**Fig. 1:**
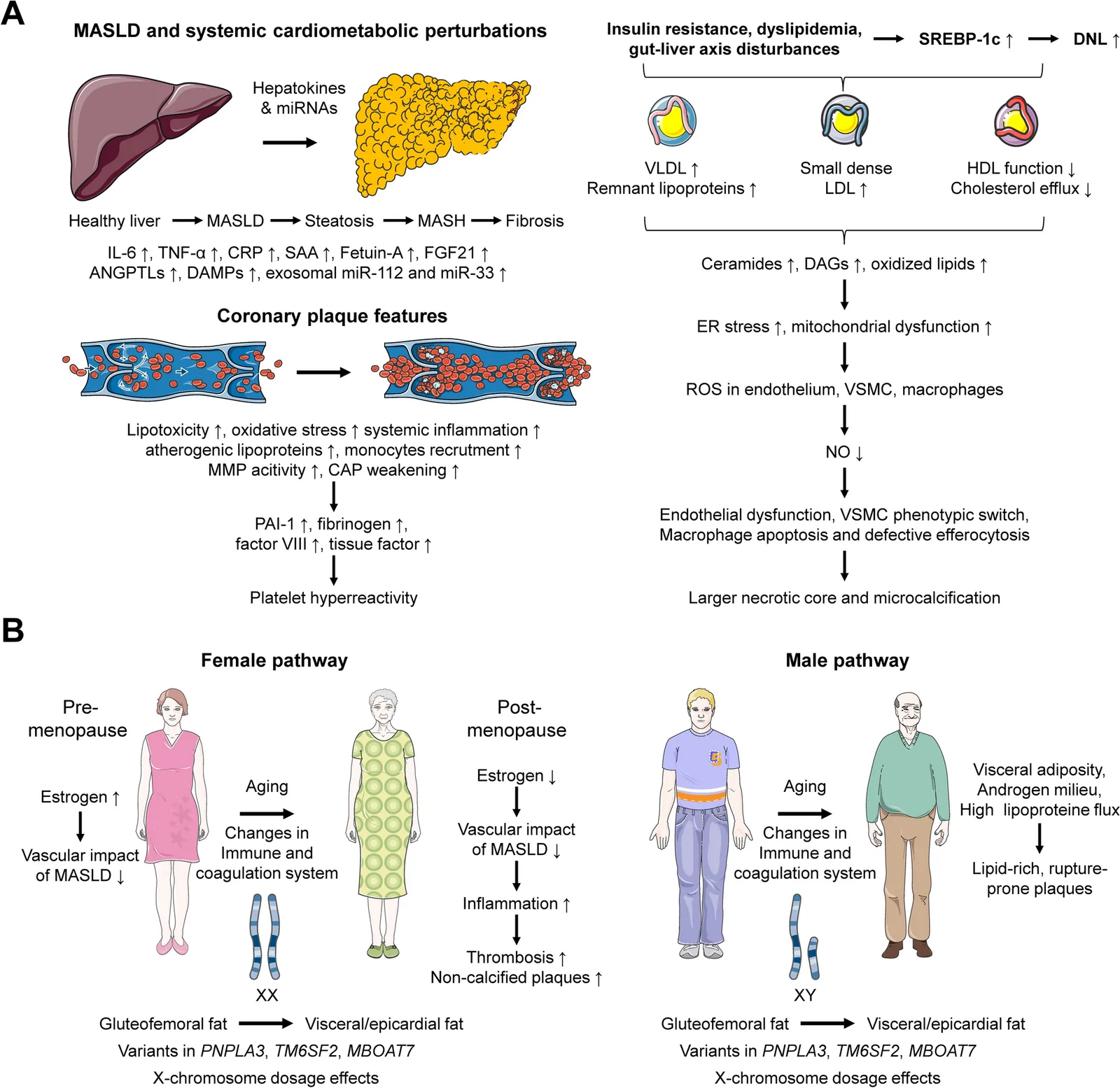
Sex-stratified pathways linking MASLD with vulnerable coronary plaque. **(A)** MASLD promotes coronary atherosclerosis through interconnected metabolic, inflammatory, lipotoxic, thrombotic, adipose-derived, and gut–liver axis pathways, leading to lipid accumulation, necrotic core expansion, fibrous cap thinning, and plaque vulnerability. **(B)** These pathways are differentially modulated by biological sex through differences in sex hormones, adipose distribution, immune and inflammatory responses, coagulation profile, and genetic/epigenetic regulation, resulting in distinct plaque phenotypes between men and women. In men, visceral adiposity and androgen-related metabolic effects favor earlier development of lipid-rich, rupture-prone plaques. In pre-menopausal women, estrogen dampens the vascular impact of MASLD, whereas after menopause, loss of estrogen is associated with a shift toward non-calcified and functionally adverse coronary disease even in the presence of lower CAC burden. *Abbreviations:* ANGPTLs, angiopoietin-like proteins; CAC, coronary artery calcium; CRP, C-reactive protein; ER, endoplasmic reticulum; FGF21, fibroblast growth factor-21; HDL, high-density lipoprotein; IL-6, interleukin-6; LDL, low-density lipoprotein; MASH, metabolic dysfunction-associated steatohepatitis; MASLD, Metabolic dysfunction-associated steatotic liver disease; MBOAT7, membrane-bound-O-acyltransferase 7; miR, micro RNA; MMP, matrix metalloproteinase; NO, nitric oxidase; PAI-1, plasminogen1 inhibitor; PNPLA3, patatin-like phospholipase domain-containing protein 3; ROS, reactive oxygen species; SAA, serum amyloid A; TM6SF2, transmembrane 6 superfamily member 2; VLDL, very low-density lipoprotein; VSMC, vascular smooth muscle cell. This image was created using Servier Medical Art, which allows to share freely professional-quality illustrations under the CC BY 4.0 license

MASLD promotes a multisystem proatherogenic cardiometabolic milieu through highly interconnected metabolic, lipotoxic, inflammatory, thrombotic, and gut-derived pathways. Biological sex modulates each pathway, leading to distinct patterns of disease expression in men and women (Fig. 1). The following subsections aim to integrate these mechanisms in a pathway-oriented, sex-modulated framework.

### Insulin resistance and atherogenic lipid remodeling

MASLD-associated insulin resistance promotes hepatic de novo lipogenesis via SREBP-1c activation, leading to overproduction of triglyceride-rich VLDL particles and accumulation of atherogenic remnant lipoproteins [8, 19–22]. Cholesteryl ester transfer protein (CETP)-mediated lipid exchange facilitates the formation of small dense LDL, while concurrent reductions in HDL impair cholesterol efflux and foam-cell clearance [8, 23]. Together, these abnormalities accelerate lipid deposition within the arterial wall, thereby promoting the expansion of the necrotic core and thinning of the fibrous cap.

In premenopausal women, estrogen attenuates hepatic lipogenesis, enhances LDL receptor activity, and maintains a less atherogenic lipoprotein profile [24]. After menopause, loss of estrogen removes this metabolic restraint, leading to a shift toward atherogenic dyslipidemia [25]. In men, physiological testosterone levels contribute to preserving insulin sensitivity and lipid partitioning, whereas testosterone deficiency promotes visceral adiposity and metabolic dysfunction [13]. In women with androgen excess (e.g., polyendocrine metabolic ovary syndrome, PMOS, previously known as polycystic ovary syndrome, PCOS), steatotic liver disease (SLD) and insulin resistance are further amplified [13]. These divergent trajectories likely contribute to earlier plaque formation in men, whereas women exhibit a sharper postmenopausal acceleration in lipid-driven atherogenesis.

### Hepatic inflammation, immune activation, and plaque instability

MASLD fosters systemic inflammation through the hepatic release of cytokines, chemokines, acute-phase reactants, hepatokines, extracellular vesicles, and damage-associated molecular patterns (DAMPs), thereby promoting monocyte recruitment and macrophage activation within the vascular wall [26–28]. These inflammatory signaling pathways promote matrix metalloproteinase (MMP) activation and extracellular matrix degradation, weakening fibrous caps and increasing plaque vulnerability [27, 28]. Progression from uncomplicated SLD to advanced liver fibrosis (F3–F4) amplifies systemic inflammation and atherogenic risk [5, 7, 29, 30].

Estrogen suppresses inflammatory signaling, macrophage activation, and macrophage MMP expression in premenopausal women, thereby supporting plaque stability [31, 32]. After menopause, loss of estrogen shifts the balance toward sustained inflammatory activity and impaired inflammatory resolution [33]. Women generally exhibit stronger innate and adaptive immune responses, including enhanced toll-like receptor (TLR) and interferon signaling [34], whereas men show earlier monocytosis and foam-cell formation [35]. These pathobiological differences likely influence both the temporal development and the magnitude of plaque inflammatory burden across sexes, although MASLD-specific evidence remains limited.

### Lipotoxicity, endothelial dysfunction, and vascular remodeling

Lipotoxic mediators such as ceramides, diacylglycerols, and oxidized lipids, among others, induce oxidative stress, mitochondrial dysfunction, endothelial injury, and phenotypic switching of vascular smooth muscle cells [36]. Collectively, these processes reduce nitric oxide bioavailability, impair efferocytosis, and promote microcalcification and necrotic core expansion. In premenopausal women, estrogen preserves endothelial homeostasis by sustaining nitric oxide synthase activity and constraining oxidative stress [31, 32]. With menopause, this vasculoprotective effect is lost, thereby heightening susceptibility to lipotoxicity-induced vascular injury [37]. In men, earlier exposure to visceral adiposity–driven lipotoxicity may accelerate endothelial dysfunction and plaque progression, whereas in women these changes emerge later but intensify after menopause [38, 39].

### Prothrombotic state and plaque destabilization

MASLD is characterized by a prothrombotic milieu with elevated PAI-1, fibrinogen, factor VIII, and tissue factor, leading to increased thrombin generation and platelet activation [3, 40]. These changes favor intraplaque hemorrhage, neovascularization, and recurrent micro thrombosis, all of which accelerate necrotic core expansion and fibrous cap thinning.

Estrogen exerts vasculoprotective and antithrombotic effects through endothelial and platelet-mediated mechanisms [19], whereas hypoestrogenic states favor a shift toward a procoagulant *milieu* [41]. Men generally exhibit greater basal thrombin generation and platelet reactivity [42, 43], which may act in concert with MASLD-associated coagulation abnormalities to potentiate thrombotic propensity. Collectively, these sex-related differences may modulate susceptibility to intraplaque hemorrhage and accelerate plaque destabilization.

### Adipose tissue-vascular crosstalk and coronary microvascular dysfunction

Epicardial and visceral adipose tissue act as active endocrine organs, releasing cytokines, adipokines, and proteases that promote endothelial activation, monocyte recruitment, and extracellular matrix degradation [44, 45]. Perivascular adipose dysfunction further impairs vasomotor regulation, thereby amplifying local vascular injury [46]. MASLD-associated metabolic and inflammatory abnormalities may also be associated with coronary microvascular disease (CMD), characterized by impaired coronary flow reserve, endothelial dysfunction, and microvascular remodeling [47, 48]. CMD is a key contributor to ischemia with non-obstructive coronary arteries (INOCA), which is more frequently observed in women [49].

Sex-related differences in adipose tissue distribution may further modulate these associations. Men typically accumulate visceral and epicardial adiposity earlier in life, whereas women experience a shift toward central and epicardial fat accumulation postmenopausally [46]. This transition may contribute to both plaque progression and CMD, potentially explaining persistent ischemic symptoms despite low or modest CAC burden in some women [33, 46, 50–52].

### Gut-liver axis dysfunction and systemic vascular inflammation

Gut dysbiosis and increased intestinal permeability in MASLD promote endotoxemia and TLR4 activation, while microbial metabolites such as Trimethylamine N-oxide (TMAO) enhance foam-cell formation, platelet reactivity, and endothelial dysfunction [8, 53, 54]. Disruption of bile acid signaling (FXR/TGR5) further contributes to vascular inflammation and calcification [3].

Estrogen may preserve gut barrier integrity and restrain inflammatory signaling, whereas post-menopasaul hypoestrogenemia could amplify endotoxin-driven vascular inflammation [55]. Although direct evidence in MASLD-related atherosclerosis remains limited, this axis may contribute to sex-specific differences in systemic vascular inflammatory burden.

### Genetic, epigenetic, and X-chromosome–linked regulation

Established genetic variants such as Patatin-like phospholipase domain-containing protein 3 (*PNPLA3*), Transmembrane 6 Superfamily Member 2 (*TM6SF2*), and Membrane-bound O-acyltransferase domain-containing protein 7 (*MBOAT7*) influence hepatic lipid metabolism and fibrosis risk [11, 56], while epigenetic regulators and microRNAs (e.g., miR-122) modulate macrophage and vascular smooth muscle cell behavior [57, 58]. These pathways may interact with hormonal status to produce sex-dependent vascular effects.

Beyond autosomal loci, emerging evidence suggests that X-chromosome gene dosage, through escape from X-inactivation, may contribute to sex differences in immune and metabolic pathways [59, 60] and may influence susceptibility to atherosclerosis independently of circulating sex hormones [61–63]. Proposed mechanisms include altered regulation of metabolic, epigenetic, and innate immune pathways [59, 64]. However, the contribution of X-chromosome dosage to MASLD-related atherosclerosis remains largely speculative, and direct evidence linking these mechanisms to coronary plaque phenotype or cardiovascular outcomes in MASLD is scarce, underscoring an important priority for future research.

## Gender-related determinants of MASLD-related atherosclerosis

Gender-related factors may also shape the development and clinical expression of MASLD-related ASCVD through their influence on lifestyle, environmental exposures, and healthcare utilization [65]. Differences in diet, physical activity, smoking, alcohol use, sleep patterns, occupational exposures, and psychosocial stress may collectively modulate cardiometabolic risk, hepatic disease severity, and downstream vascular injury [11, 15]. In many settings, men are more frequently exposed to smoking- and alcohol-related risk behaviors, whereas women may face barriers to sustained physical activity and greater caregiving responsibilities, both of which may adversely affect metabolic health [66, 67].

Additionally, gender may shape healthcare-seeking behavior, disease recognition, and treatment patterns. Although women are generally more engaged in preventive healthcare [68], they may nonetheless experience under-recognition of cardiovascular symptoms and lower use of evidence-based therapies, including statins [15]. In parallel, the growing population of transgender individuals and the use of gender-affirming hormone therapy may introduce additional metabolic and cardiovascular determinants that could influence MASLD-related ASCVD [11, 69, 70]. Overall, however, evidence regarding the contribution of gender-related determinants to MASLD-related atherosclerosis remains limited, suggesting the necessity of additional and more focused research.

## Sex-specific plaque phenotype and cardiovascular outcomes in MASLD

The relationship between MASLD and ASCVD differs significantly between men and women in terms of plaque burden, composition, and clinical outcomes (Table 1). Several imaging studies support the idea that MASLD in men is more likely to be linked with calcified and obstructive coronary atherosclerosis. In a cohort of 8,705 individuals, MASLD was associated with CAC only in non-obese men, with no significant association observed in women [71]. Similarly, among individuals undergoing coronary computed tomography angiography (CCTA), moderate-to-severe MASLD was strongly linked to severe CAC (CAC ≥ 300), and both single-vessel and multivessel coronary calcification in men, but not in women [9]. Sex-stratified machine learning models have also suggested that MASLD contributes more strongly to CAC prediction in men than in women [72]. Furthermore, in postmenopausal women, MASLD was not an independent predictor of CAC [73]. This apparent sex-specific calcification pattern may extend beyond the coronary circulation and has also been observed for abdominal aortic calcification (AAC); however, the available evidence remains inconsistent, with some studies reporting stronger associations in women and others demonstrating a higher risk in men [74, 75].

**Table 1:** Studies assessing sex-specific associations between MASLD and atherosclerotic plaque phenotype, vascular dysfunction, and cardiovascular outcomes

Author, year [Ref]	Atherosclerotic burden	Method	Sex-specific findings	Sex-specific conclusions
Kim et al., 2015 [73]	CAC	Among 4,377 participants who underwent cardiac computed tomography in a health promotion center, 919 postmenopausal women were enrolled. Anthropometric profiles and multiple CVR factors were measured. NAFLD was measured by ultrasonography, and CAC was evaluated by cardiac computed tomography. Odds ratios and 95% CI for the presence of CAC, by severity of fatty liver disease, were estimated using logistic regression	Women were stratified into three groups by severity of NAFLD. There were significant differences in cardiovascular parameters among the groups, and prevalence of CAC significantly increased with severity of NAFLD. On logistic regression analysis after adjustment for multiple risk factors, the odds ratios for the prevalence of CAC were as follows (P < 0.05): no NAFLD, 1.0; mild NAFLD, 1.34 (95% CI, 0.92–2.16); moderate to severe NAFLD, 1.83 (95% CI, 1.06–3.16). However, this association was attenuated after adjustment for insulin resistance (P = 0.16)	There is a significant correlation between MASLD and prevalence of CAC, but MASLD is not an independent factor for coronary atherosclerosis in postmenopausal women
Kim et al., 2020 [71]	CAC	This study evaluated whether NAFLD was associated with CAC in individuals without a prior history of CVD and whether this association differed according to sex and obesity status after adjustment for traditional atherosclerotic risk factors, alcohol intake, and liver enzyme levels. Among 67,441 participants, data from 8,705 individuals who underwent assessments of fatty liver status and CAC during routine health screening were analyzed. CAC scores were calculated using computed tomography, and NAFLD was diagnosed based on evidence of hepatic steatosis detected by ultrasonography	Subgroup analysis revealed a significant association between NAFLD and CAC in non-obese male participants (OR 1.36, 95% CI 1.07–1.75), but not in female participants	MASLD is associated with the CAC only in men, but not in women
Lankarani et al., 2025 [9]	CAC	Patients without previous CVD disease were prospectively recruited. Liver fat was assessed via ultrasonography and categorized as mild or moderate-to-severe. CKD was classified using the eGFR. CAC burden was quantified as 0, 1–299, ≥ 300, single-vessel, or multivessel with coronary computed tomography. Stepwise backward multinomial logistic regression was applied for analysis	A total of 446 patients (59.2% female, average age 52.9 years) were included In subgroup analysis, Moderate-to-severe MASLD was associated with CAC 1–299 (aOR 4.07, 95% CI 1.56 –10.62), CAC ≥ 300 (aOR 8.38, 95% CI 1.55–45.32), Single vessel CAC (aOR 3.76, 95% CI 1.30 – 10.90), and multivessel CAC (aOR 4.58, 95% CI 1.56 – 13.45) in men. However, MASLD was not associated with either the absolute value of CAC or its distribution in women	MASLD is associated with CAC score and CAC distribution in men, but not in women
Jamalinia et al., 2026 [72]	CAC	In this cross-sectional study, adults referred for CCTA were subdivided into training (127 men, 185 women) and test (55 men, 79 women) sets. Eight Machine learning classifiers were trained separately for men and women to predict CAC presence (0 vs. > 0), severity (0, 1–99, ≥ 100), and distribution (0, 1-vessel, ≥ 2-vessel)	Feature importance analysis of machine learning algorithms demonstrated substantial sex-related differences in predictor ranking, with MASLD contributing more prominently to CAC prediction models in men than in women	MASLD appears to have greater predictive relevance for CAC burden and distribution in men compared with women
Mellinger et al., 2015 [74]	CAC and AAC	Hepatic steatosis was measured in 3529 participants using multidetector computed tomography scanning. Multivariable logistic regression was used to determine whether hepatic steatosis is associated with prevalent CVD adjusted for covariates. It also tested whether associations were independent of other metabolic diseases/traits. Subclinical cardiovascular outcomes were CAC and AAC	When stratified by sex, the association between hepatic steatosis and AAC was strongest in men (aOR 1.26, 95% CI 1.13–1.39) than in women (aOR 1.03, 95% CI 1.03 – 1.17), whereas no such sex difference was noted for CAC (aOR 1.20, 95% CI 1.08–1.33 in men; aOR 1.19, 95% CI 1.04–1.36 in women)	SLD was associated with CAC in both sexes, whereas its association with AAC was observed only in men
Remigio-Baker et al., 2017 [75]	AAC	Total of 1004 adults from the Multi-Ethnic Study of Atherosclerosis were studied to assess the relationship between NAFLD (liver-to-spleen ratio < 1) and the following measures of AAC: presence (volume score > 0, using Poisson regression); change in volume score (increasing vs. no change, using Poisson regression); and morphology (volume and density score, where volume score > 0, using linear regression); and interaction by race/ethnicity and sex	MASLD was associated with abdominal aortic calcification in women (aOR 1.13, 95% CI 1.00–1.27) but not in men (aOR 1.00, 95% CI 0.87–1.15)	MASLD was associated with abdominal aortic calcification only in women
Kim et al., 2013 [76]	CIMT	This was a cross-sectional analysis of 769 healthy individuals (326 men and 443 women) with an ALT concentration ≤ 40 IU/L and alcohol consumption < 140 g/week. Mean CIMT was measured using ultrasound. NAFLD was defined as a mild or greater degree of hepatic steatosis on ultrasound	Although all subjects had an ALT concentration within the reference range, the prevalence of NAFLD increased with increasing quartiles of ALT concentration (27.1%, 40.0%, 54.7%, 75.3% in men, P for trend < 0.001; 22.0%, 34.4%, 35.7%, 55.0% in women, P for trend < 0.001). In the 3rd and 4th quartiles of ALT concentration, women with NAFLD had a significantly higher C-IMT than those without NAFLD (0.671 ± 0.019 mm vs. 0.742 ± 0.025 mm, P = 0.023 in Q3; 0.651 ± 0.023 mm vs. 0.737 ± 0.021 mm, P = 0.005 in Q4). These differences remained significant even after adjusting for a broad spectrum of potential confounders. In contrast, although men with NAFLD tended to have a higher C-IMT than those without NAFLD in each quartile, these differences were not statistically significant	MASLD was independently associated with CIMT in women but not men
Martínez-Alvarado et al., 2014 [77]	CIMT	This study included 846 subjects from the Genetic of Atherosclerosis Disease (GEA) study (53 ± 9 years, 50.7% women) without diabetes and no personal or family history of premature CAD. Blood samples were taken for measurements of lipids profile, uric acid, and insulin. The presence of FL was identified by computed tomography. CIMT was measured by B mode ultrasound, using the > 75 percentile as cutoff value to define subclinical atherosclerosis	The general prevalence of FL was 32.4%. In women, FL was associated with a 66% higher probability of having high CIMT, independently of age, hypertension, dyslipidemia, and waist circumference, but not of HOMA-IR	SLD was associated with CIMT in women but not in men
Kim et al., 2016 [78]	CIMT	Patients who had undergone carotid and abdominal ultrasound between June 2011 and December 2013 were retrospectively evaluated. The differences between the CIMT values measured in the common carotid artery and the prevalence of carotid plaque in patients with fatty liver disease and those with normal livers were investigated	In binary regression analysis, MASLD was associated with increased carotid intima media thickness in women (aOR 1.65, 95% CI 1.01–2.69) but not in men (OR 1.05, 95% CI 0.76–1.44)	MASLD is associated with the CIMT only in women, but not in men
Li et al., 2017 [79]	CIMT, cf-PWV, and FMD	1007 postmenopausal women were divided into two groups (NAFLD and controls) and then three groups (NAFLD with metabolic syndrome, NAFLD without metabolic syndrome, and controls), respectively. To exclude the influence of confounding factors, we studied a specifically selected group with no additional CVR. CIMT, cf-PWV, and FMD were measured in all study patients and controls	Patients with NAFLD had significantly greater CIMT than did controls (P < 0.001). cf-PWV was also significantly higher in patients with NAFLD than in controls (P < 0.001). We found significantly decreased FMD in patients with NAFLD than in control patients (P < 0.001). The observed association was independent of the presence of metabolic syndrome	In postmenopausal women MASLD was associated with increased arterial stiffness and carotid intima media thickness, and decreased flow-mediated dilatation
Yoo et al., 2024 [80]	baPWV	Participants who had health examinations between 2006 and 2019 were analyzed for fatty liver and increased baPWV using liver ultrasonography and automatic volume plethysmography device. Participants were classified based on presence of MAFLD or NAFLD and further divided into subgroups: no fatty liver disease (reference), NAFLD-only, MAFLD-only, and both NAFLD and MAFLD. Subgroups were additionally stratified by sex. Cox proportional hazard model was utilized to analyze the risk of developing baPWV ≥ 1400 cm/s in participants without baseline elevation of the baPWV	Incidence of NAFLD or MAFLD were higher in men than in women but aHR of developing the increase in AS was higher in women. In subgroup analysis, the MAFLD-only group presented the strongest associations with increase in AS (aHR, 1.53 [95% CI, 1.43–1.64]), with the trend more pronounced in women than in men (Women, aHR, 1.63 [95% CI, 1.08–2.46]; Men, aHR 1.45 [95% CI, 1.35–1.56])	The associations between MASLD and arterial stiffness, an indicator of atherosclerosis, is stronger in women than in men
Henney et al., 2024 [14]	Microvascular events	Retrospective cohort study using the TriNetX global database comparing patients with hepatic steatosis plus ≥ 1 metabolic syndrome component vs matched controls without steatosis or MetS. Propensity score matching (1:1) was applied with 5-year follow-up. Outcome included microvascular (neuropathy, retinopathy, nephropathy) events	Compared to the matched reference arm, MASLD was associated with a higher risk of microvascular events in women (aHR 7.01, 95% CI 5.46–9.00) than in men (aHR 4.26, 95% CI 3.37– 5.38). This higher risk in women was consistently observed in each pair of datasets	The associations between MASLD and microvascular disease is stronger in women than in men
Jamalinia et al., 2025 [6]	fatal and/or non-fatal CVD events	Four major electronic databases were searched from inception to November 2024 to identify observational cohort studies examining sex-specific associations between MASLD and the risk of fatal and/or non-fatal CVD events. The diagnosis of MASLD and its severity were assessed using serum biomarkers/scores, International Classification of Diseases codes, imaging or histology	Thirty-six cohort studies with aggregate data on ~ 18.5 million individuals were included (~ 25% with MASLD; 48% women; mean age of 50.2 years). During a median follow-up of 6.9 years (IQR 5.0–12.3), approximately 515 000 fatal and/or non-fatal CVD events occurred (42% in women). MASLD was associated with a higher risk of fatal or non-fatal CVD events in women (pooled HR 1.59, 95% CI 1.44–1.75; I2 = 96.10%) than in men (pooled HR 1.37, 95% CI 1.27–1.48; I2 = 96.26%) (p-value for sex difference = 0.018). The severity of MASLD (variably assessed) further increased the magnitude of this risk, especially in women (pooled HR 2.40, 95% CI 1.73–3.32; I2 = 57.29%). In men, although MASLD severity was associated with an increased risk of CVD events, this association did not achieve statistical significance (pooled HR for MASLD severity: 1.69, 95% CI 1.21–2.35, I^2^ = 78.67%) when compared to the overall MASLD-related CVD risk in men	Women with MASLD are at higher risk of incident fatal and non-fatal CVD events compared with men, especially as the severity of MASLD increases

AAC, abdominal aortic calcification; aHR, adjusted hazard ratio; ALT, alanine aminotransferase; aOR, adjusted odds ratio; AS, arterial stiffness; baPWV, brachial-ankle pulse wave velocity; CAC, coronary artery calcium; CAD, coronary artery disease; cf-PWV, carotid-femoral pulse wave velocity; CI, confidence interval; CIMT, carotid intima-media thickness; CCTA, coronary computed tomography angiography; CKD, chronic kidney disease; CVD, cardiovascular disease; CVR, cardiovascular risk; eGFR, estimated glomerular filtration rate; FMD, flow-mediated dilatation; FL, fatty liver; HOMA-IR, homeostasis model assessment of insulin resistance; HR, hazard ratio; MAFLD, metabolic dysfunction-associated fatty liver disease; MASLD, metabolic dysfunction-associated steatotic liver disease; MetS, metabolic syndrome; NAFLD, non-alcoholic fatty liver disease; OR, odds ratio; SLD, steatotic liver disease.

In contrast, MASLD in women appears to be associated with non-calcified atherosclerotic phenotypes and functional markers of vascular dysfunction. Independent associations between MASLD and carotid intima-media thickness (CIMT) have been reported predominantly in women, even after adjustment for traditional cardiometabolic risk factors [76–78]. Beyond structural vascular abnormalities, MASLD in postmenopausal women has been associated with increased arterial stiffness, higher carotid-femoral pulse wave velocity, and impaired flow-mediated dilatation independent of metabolic syndrome [79]. Longitudinal data further demonstrate that the relationship between MASLD and progression of arterial stiffness was stronger in women than in men [80].

Further support for this sex-specific paradigm comes from a large real-world cohort study, which found that MASLD was associated with a higher risk of microvascular events in women than in men (adjusted Hazard Ratio [HR] 7.01 vs. 4.26) [14]. This finding extends MASLD-associated vascular injury beyond the macrovascular domain and demonstrates that women may exhibit a disproportionate susceptibility to microvascular dysfunction. Additionally, a large meta-analysis including approximately 18.5 million individuals, showed that MASLD was linked to a significantly higher risk of fatal and/or non-fatal cardiovascular events in women than in men (pooled HR 1.59 vs. 1.37, respectively) [6]. The impact of MASLD severity (variably assessed) on cardiovascular events also appeared to be greater in women than in men (pooled HR 2.40 vs. 1.69, respectively) [6].

**Fig. 2:**
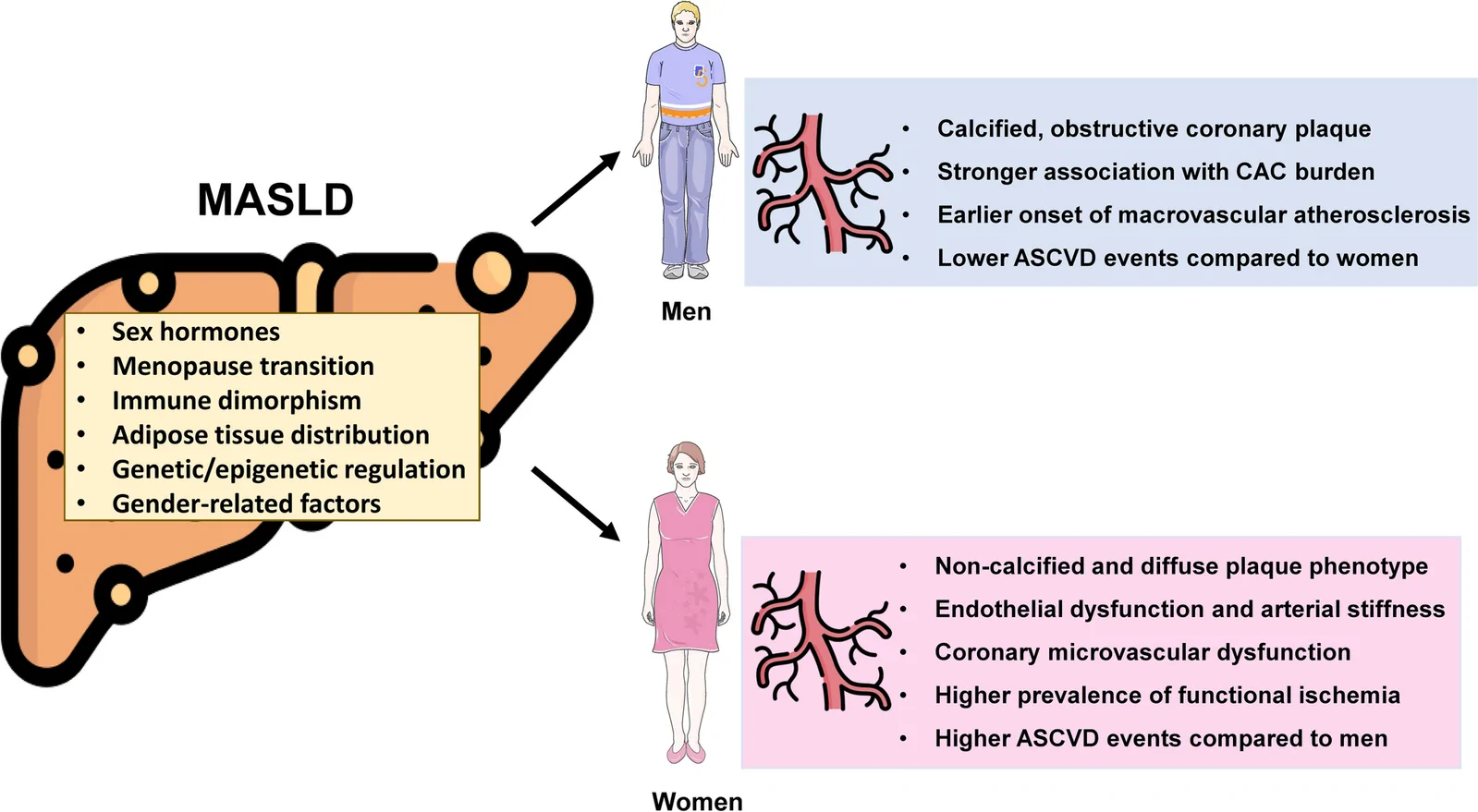
Sex-specific differences in MASLD-related atherosclerotic phenotype and cardiovascular outcomes. The figure summarizes the distinct vascular patterns associated with MASLD in men and women. In men, MASLD is more consistently associated with increased CAC burden. In contrast, women demonstrate weaker associations with CAC but stronger associations with non-calcified atherosclerotic phenotypes and functional vascular abnormalities. Despite having a lower calcific plaque burden, women with MASLD appear to face a disproportionately higher risk of ASCVD events. These differences are modulated by sex-related factors, including sex hormones, menopausal transition, immune responses, adipose distribution, genetic and epigenetic regulation, and gender-related determinants. *Abbreviations:* ASCVD, atherosclerotic cardiovascular disease; CAC, coronary artery calcium; CIMT, carotid intima-media thickness; CVD, cardiovascular disease; MASLD, metabolic dysfunction-associated steatotic liver disease

These observations support the idea that MASLD-related vascular disease in women is not simply characterized by “less calcification,” but rather by a shift toward functional and microvascular pathology. Evidence from the CAC Consortium also shows higher relative cardiovascular mortality among women than men at comparable CAC levels [81]. Moreover, CAC density appears less predictive of ASCVD outcomes in women [81]. Collectively, data support the notion that calcium-centric assessment may systematically underestimate ASCVD risk in women with MASLD by failing to capture the combined high-risk phenotype of non-calcified plaque, endothelial dysfunction, and CMD (Fig. 2). Consideration of sex discrepancies further underscores the importance of a multimodality vascular assessment framework encompassing both macrovascular and microvascular domains.

## Knowledge gaps and future directions

Despite growing evidence that men and women exhibit distinct patterns of MASLD-related atherosclerosis, several important knowledge gaps limit current understanding. First, much of the available evidence is derived from observational and predominantly cross-sectional studies, restricting causal inference regarding the temporal relationship between MASLD progression, plaque evolution, and cardiovascular outcomes.

In addition, substantial heterogeneity exists in the definition and staging of MASLD, with studies relying on varying combinations of ultrasonography, computed tomography, non-invasive fibrosis scores, administrative coding, or histology. Cardiovascular phenotypes have likewise been assessed inconsistently across studies using CAC, CIMT, arterial stiffness, endothelial function, CCTA, or clinical outcomes, which limit direct comparability.

Another major limitation of the current literature is the reliance on calcification-centered assessments. Although CAC is a validated marker of atherosclerotic burden, it does not adequately capture non-calcified plaque, vascular inflammation, endothelial dysfunction, or CMD [82]. This limitation may be particularly relevant in women, who frequently demonstrate lower calcific burden but a higher risk of MASLD-related ASCVD events. Whether these observations reflect fundamentally different plaque phenotypes, differences in disease progression, or limitations of current imaging approaches remains uncertain.

Several biological mechanisms proposed to explain sex differences in MASLD-related atherosclerosis also require further validation. While the roles of sex hormones, adipose distribution, immune dimorphism, and metabolic dysfunction are supported by substantial evidence, the contribution of genetic, epigenetic, and X-chromosome-related pathways remains largely hypothetical.

The generalizability of current findings also remains uncertain. Many of the available cohorts originate from the Middle East or East Asia, and sex-specific associations may differ across ethnic, genetic, environmental, and sociocultural contexts. Consequently, it remains unclear whether the vascular phenotypes described in current studies are broadly applicable across diverse populations.

Another important but underappreciated area is the contribution of gender-related determinants. Lifestyle behaviors, healthcare utilization, socioeconomic factors, health literacy, preventive treatment patterns, and gender-related differences in symptom recognition may substantially influence ASCVD risk and outcomes in MASLD. However, these variables are rarely incorporated into existing studies, making it challenging to distinguish between biological sex effects and sociocultural influences.

**Fig. 3:**
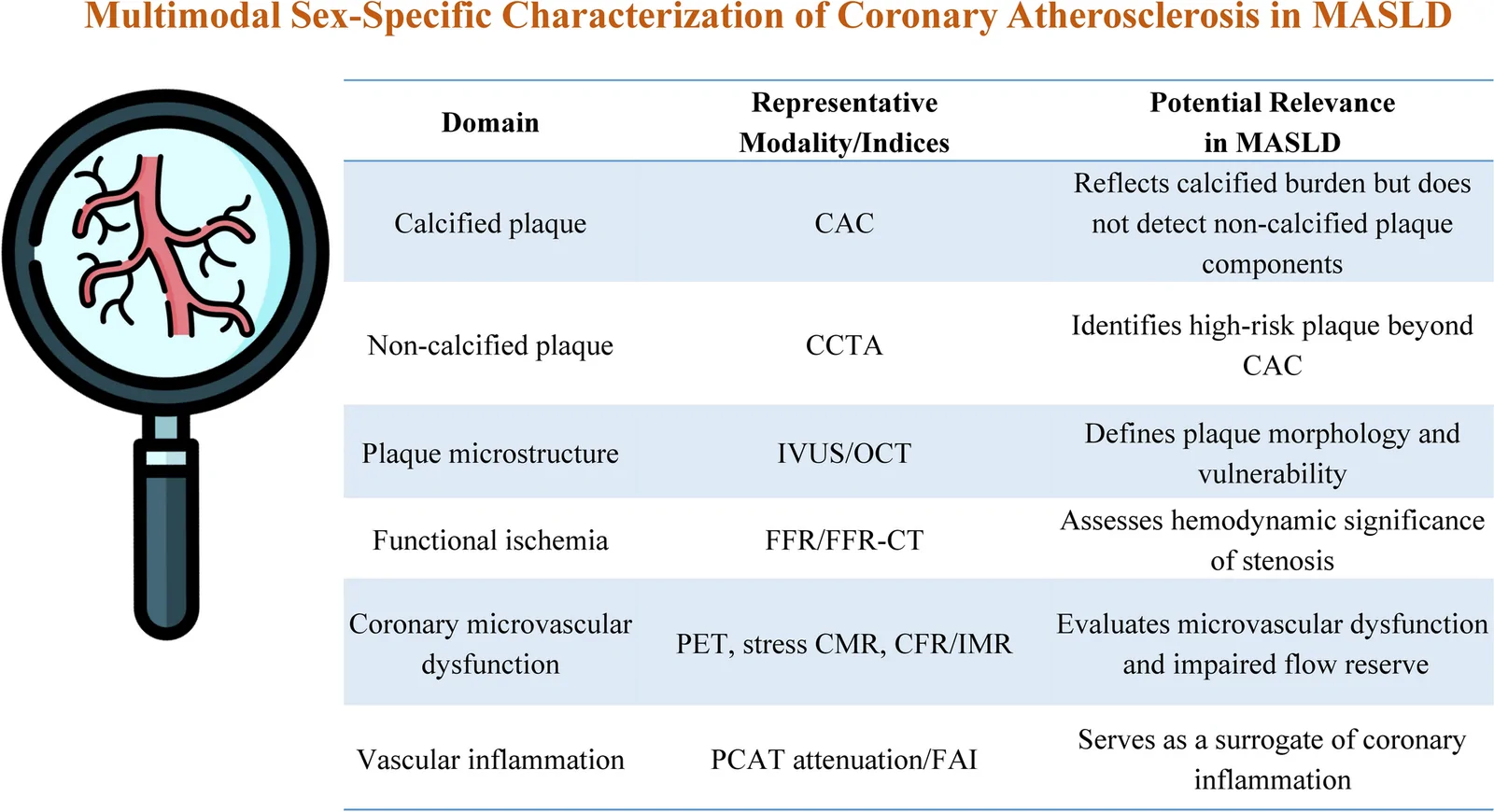
Multimodality imaging framework for the sex-specific characterization of coronary atherosclerosis in MASLD. The figure illustrates a complementary imaging framework for characterizing MASLD-related coronary disease across structural, functional, and inflammatory domains. Importantly, calcification-centered assessment (e.g., CAC) may underestimate clinically relevant disease in women with MASLD. Accordingly, CCTA and functional/inflammatory imaging modalities may provide essential complementary information for accurate ASCVD risk stratification beyond CAC. *Abbreviations:* ASCVD, atherosclerotic cardiovascular disease; CAC, coronary artery calcium; CCTA, coronary computed tomography angiography; IVUS, intravascular ultrasound; OCT, optical coherence tomography; FFR, fractional flow reserve; FFR-CT, computed tomography-derived fractional flow reserve; PET, positron emission tomography; CMR, cardiac magnetic resonance; CFR, coronary flow reserve; IMR, index of microcirculatory resistance; PCAT, pericoronary adipose tissue; FAI, fat attenuation index

**Fig. 4:**
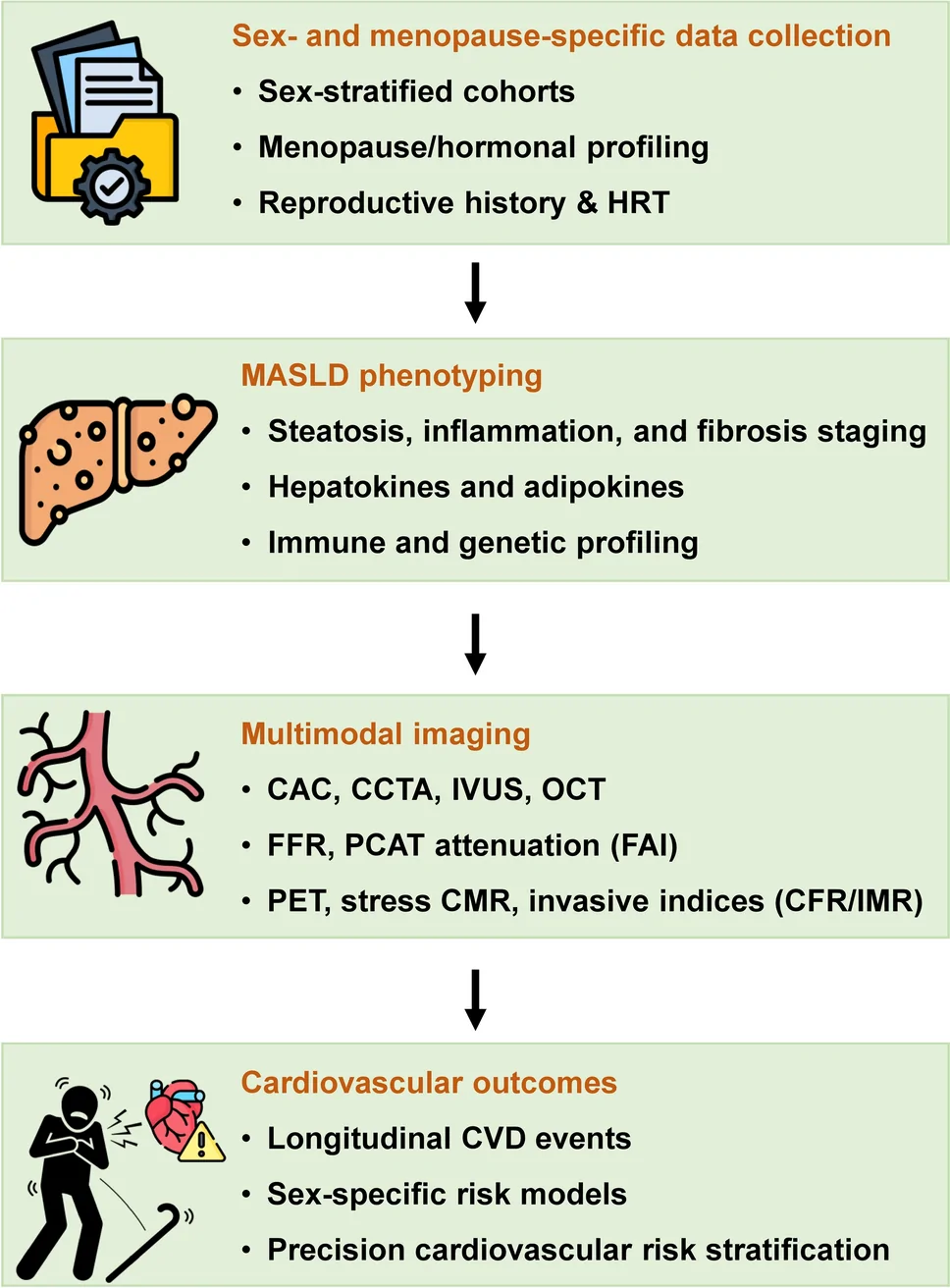
Proposed framework for future research on sex-specific MASLD-related atherosclerotic phenotypes. This schematic summarizes key research priorities to advance understanding of sex-specific cardiovascular risk in MASLD. Studies should incorporate sex- and menopause-specific data, standardized MASLD phenotyping, multimodality imaging to capture plaque burden, composition, ischemia, remodeling, and inflammation, and associate these imaging-derived phenotypes with longitudinal cardiovascular outcomes to enable sex-specific risk prediction and precise ASCVD risk stratification. *Abbreviations:* ASCVD, atherosclerotic cardiovascular disease; CAC, coronary artery calcium; CCTA, coronary computed tomography angiography; IVUS, intravascular ultrasound; OCT, optical coherence tomography; FFR, fractional flow reserve; FFR-CT, computed tomography-derived fractional flow reserve; PET, positron emission tomography; CMR, cardiac magnetic resonance; CFR, coronary flow reserve; IMR, index of microcirculatory resistance; PCAT, pericoronary adipose tissue; MASLD, metabolic dysfunction-associated steatotic liver disease; FAI, fat attenuation index.

Addressing these limitations will require multinational studies that adopt prespecified sex-stratified designs and systematically capture menopausal status, reproductive history, hormone therapy, androgen status, and relevant gender-related variables. In parallel, standardized characterization of MASLD severity and incorporation of mechanistic biomarkers may clarify the biological pathways linking MASLD to sex-specific ASCVD risk. Finally, multimodality cardiovascular phenotyping (Fig. 3) should move beyond calcification alone [83–87]. Future investigations should evaluate how plaque composition, vascular inflammation, endothelial dysfunction, and CMD contribute to cardiovascular outcomes in men and women with MASLD. Linking these imaging-derived phenotypes to longitudinal cardiovascular events will be essential for determining whether distinct vascular phenotypes translate into different clinical trajectories and for developing sex-informed approaches to ASCVD risk stratification and prevention in MASLD (Fig. 4).

## Conclusion

MASLD is increasingly recognized as a major contributor to ASCVD, yet its vascular manifestations differ substantially between men and women. Current data support a sex-specific paradigm in which men with MASLD more commonly develop calcified and obstructive coronary atherosclerosis, whereas women with MASLD more often exhibit non-calcified plaque and functional vascular abnormalities (e.g., endothelial dysfunction, arterial stiffness, and CMD). Importantly, this dissociation between relatively lower CAC burden and greater functional and microvascular vulnerability in women suggests that calcification alone is an incomplete marker of ASCVD severity in this population.

These differences have important clinical implications. CAC scoring should be interpreted cautiously in women with MASLD, particularly in those with persistent metabolic or inflammatory risk, as low or absent CAC does not exclude clinically relevant atherosclerotic or CMD. In selected high-risk women (e.g., those with symptoms, high cardiometabolic risk, or discordant clinical profiles), complementary evaluation with CCTA and functional or microvascular testing may improve risk stratification and guide earlier preventive intervention, including more proactive management of cardiometabolic risk factors and evidence-based therapies such as statins.

Future studies should also adopt sex- and menopause-stratified designs with standardized MASLD phenotyping and longitudinal multimodality imaging linked to cardiovascular outcomes to enable more precise, sex-informed ASCVD prevention strategies.

## Supplementary Information

Additional file1 (DOCX 14 kb)

## Data Availability

No datasets were generated or analysed during the current study.

## Abbreviations

ANGPTLs: Angiopoietin-like proteins
ASCVD: Atherosclerotic cardiovascular disease
CAC: Coronary artery calcium
CAD: Coronary artery disease
CC BY 4.0: Creative commons attribution 4.0 international license
CCTA: Coronary computed tomography angiography
CETP: Cholesteryl ester transfer protein
CFR: Coronary flow reserve
CMD: Coronary microvascular disease
CIMT: Carotid intima-media thickness
CMR: Cardiac magnetic resonance
CRP: C-reactive protein
CT: Computed tomography
CT-FFR: Computed tomography-derived fractional flow reserve
CVD: Cardiovascular disease
DAMPs: Damage-associated molecular patterns
EAT: Epicardial adipose tissue
ER: Endoplasmic reticulum
FAI: Fat attenuation index
FFR: Fractional flow reserve
FGF21: Fibroblast growth factor 21
FXR: Farnesoid X receptor
HDL: High-density lipoprotein
HRT: Hormone replacement therapy
ICD: International classification of diseases
IL-6: Interleukin-6
IMR: Index of microcirculatory resistance
INOCA: Ischemia with non-obstructive coronary arteries
IRAK1: Interleukin-1 receptor-associated kinase 1
IVUS: Intravascular ultrasound
KDM6A: Lysine demethylase 6A
LDL: Low-density lipoprotein
LPS: Lipopolysaccharide
MASH: Metabolic dysfunction-associated steatohepatitis
MASLD: Metabolic dysfunction-associated steatotic liver disease
MBOAT7: Membrane-bound O-acyltransferase domain-containing 7
MeSH: Medical subject headings
miR: MicroRNA
MMP: Matrix metalloproteinase
NAFLD: Nonalcoholic fatty liver disease
NO: Nitric oxide
OCT: Optical coherence tomography
PAI-1: Plasminogen activator inhibitor-1
PCAT: Pericoronary adipose tissue
PET: Positron emission tomography
PMOS: Polyendocrine metabolic ovarian syndrome
PNPLA3: Patatin-like phospholipase domain-containing protein 3
ROS: Reactive oxygen species
SAA: Serum amyloid A
SLD: Steatotic liver disease
SREBP-1c: Sterol regulatory element-binding protein 1c
TIMP1: Tissue inhibitor of metalloproteinases 1
TLR: Toll-like receptor
TMAO: Trimethylamine N-oxide
TM6SF2: Transmembrane 6 superfamily member 2
TGR5: Takeda G protein-coupled receptor 5
VLDL: Very low-density lipoprotein
VSMC: Vascular smooth muscle cell
