# Supplementary material for: Sex differences in MASLD-related atherosclerosis: plaque phenotype, vascular dysfunction, and cardiovascular outcomes
Source: Biol Sex Differ. 2026 Jul 7;17:162. doi: 10.1186/s13293-026-00952-3 (PMC13629124; doi:10.1186/s13293-026-00952-3)
Supplement: Supplementary file 1 [file 13293_2026_952_MOESM1_ESM.docx]

| **Supplementary Table 1. Search syntax used to identify studies evaluating sex differences in MASLD-related atherosclerosis burden.** | |
| --- | --- |
| **Database** | **Syntax** |
| PubMed/MEDLINE | (“non-alcoholic fatty liver disease”[Mesh] ***OR*** “NAFLD”[tiab] ***OR*** “fatty liver”[tiab] ***OR*** “nonalcoholic steatohepatitis”[tiab] ***OR*** “non-alcoholic steatohepatitis“[tiab] ***OR*** “NASH”[tiab] ***OR*** “metabolic dysfunction-associated steatohepatitis”[tiab] ***OR*** “MASH”[tiab] ***OR*** “MASLD”[tiab] ***OR*** “steatotic liver”[tiab] ***OR*** “MAFLD”[tiab]) ***AND*** (“atherosclerosis”[Mesh] ***OR*** “atherosclerosis”[tiab] ***OR*** “atherosclerotic”[tiab] ***OR*** “atherogenesis”[tiab] ***OR*** “coronary artery disease”[Mesh] ***OR*** “coronary artery disease”[tiab] ***OR*** “CAD”[tiab] ***OR*** “coronary heart disease”[tiab] ***OR*** “ischemic heart disease”[tiab] ***OR*** “ischemic cardiomyopathy”[tiab] ***OR*** “cardiovascular disease”[tiab] ***OR*** “ASCVD”[tiab] ***OR*** “coronary atherosclerosis”[tiab] ***OR*** “subclinical atherosclerosis”[tiab] ***OR*** “arterial plaque”[tiab] ***OR*** “atherosclerotic plaque”[tiab] ***OR*** “coronary plaque”[tiab] ***OR*** “plaque phenotype”[tiab] ***OR*** “plaque vulnerability”[tiab] ***OR*** “vulnerable plaque”[tiab] ***OR*** “high-risk plaque”[tiab] ***OR*** “thin-cap fibroatheroma”[tiab] ***OR*** “TCFA”[tiab] ***OR*** “low-attenuation plaque”[tiab] ***OR*** “fibroatheroma”[tiab] ***OR*** “necrotic core”[tiab] ***OR*** “positive remodeling”[tiab] ***OR*** “spotty calcification”[tiab] ***OR*** “coronary calcification”[tiab] ***OR*** “vascular calcification”[tiab] ***OR*** “coronary artery calcium”[tiab] ***OR*** “CAC”[tiab] ***OR*** “coronary stenosis”[tiab] ***OR*** “obstructive coronary disease”[tiab] ***OR*** “non-obstructive coronary disease”[tiab] ***OR*** “microvascular dysfunction”[tiab] ***OR*** “endothelial dysfunction”[tiab] ***OR*** “coronary microvascular dysfunction”[tiab] ***OR*** “intima-media thickness”[tiab] ***OR*** “carotid plaque”[tiab] ***OR*** “carotid atherosclerosis”[tiab] ***OR*** “myocardial infarction”[Mesh] ***OR*** “myocardial infarction”[tiab] ***OR*** “acute coronary syndrome”[tiab] ***OR*** “MACE”[tiab]) ***AND*** (“sex characteristics”[Mesh] ***OR*** “sex factors”[Mesh] ***OR*** “sex difference*”[Mesh] ***OR*** “sex difference*”[tiab] ***OR*** “sex-specific”[tiab] ***OR*** “gender-specific”[tiab] ***OR*** “gender differences”[tiab] ***OR*** “menopaus*”[tiab]) ***AND*** ("1900/01/01"[Date - Publication] : "2026/04/30"[Date - Publication]) ***AND*** (humans[Filter]) ***AND*** (alladult[Filter]) |
| Note: MeSH = Medical Subject Headings; [tiab] = search in title/abstract; [Date - Publication] = publication date filter; [Filter] = population filter; Boolean operators: AND = include both terms, OR = include any term; parentheses () = group terms; * (asterisk) = wildcard to capture multiple word endings; search limited to human adults, from inception through April 30, 2026. | |

***Supplementary Material***
